# MicroRNA-152 Promotes Slow-Twitch Myofiber Formation via Targeting Uncoupling Protein-3 Gene

**DOI:** 10.3390/ani9090669

**Published:** 2019-09-10

**Authors:** Yong Zhang, Honglin Yan, Pan Zhou, Zhenzhen Zhang, Jingbo Liu, Hongfu Zhang

**Affiliations:** 1 School of Life Science and Engineering, Southwest University of Science and Technology, Mianyang 621010, China; 2 State Key Laboratory of Animal Nutrition, Institute of Animal Sciences, Chinese Academy of Agricultural Sciences, Beijing 100193, China

**Keywords:** miR-152, UCP3, myofiber specification, myogenesis, meat quality, porcine myoblasts

## Abstract

**Simple Summary:**

Different pig breeds exhibit evident diversities in pork quality characteristics, which largely resulted from the differences in myofiber type compositions. To explore the decisive role of microRNAs (miRNAs) in myofiber specification, myofiber type compositions, miR-152 expression patterns in various tissues and miR-152 expression level in the longissimus dorsi (LD) muscles from either Rongchang (RC) or large white (LW) pigs were determined. We found that the longissimus dorsi (LD) muscles of the RC pigs have higher proportion of slow-twitch myofibers and more abundant level of miR-152 than those of the LW pigs, which indicated that miR-152 might be involved in myofiber type compositions and myogenesis. Then, the gain- and loss-of-function trials and luciferase activity assays confirmed that miR-152 could promote slow-twitch myofiber formation and skeletal myogenesis via targeting uncoupling protein 3 (*UCP3*) gene. The knockdown of UCP3 and rescue experiment further verified that UCP3 mediates miR-152 action in slow-twitch myofiber formation. Our findings suggested that miR-152 and its target gene *UCP3* might be feasible markers for interposition intended to promoting meat quality in domesticated animals.

**Abstract:**

The differences of pork quality characteristics among different pig breeds mainly came from the differences in myofiber type compositions. Growing evidence indicated the key role of miRNAs in myofiber specification. In the present study, we found that miR-152 is more abundant in the slow-twitch myofiber-enriched muscles. However, its role in myofiber type transformation and myogenesis is largely unknown. Overexpression of miR-152 in porcine myotubes promoted the formation of slow-twitch myofibers and myogenesis. While, inhibition of miR-152 expression showed the opposite effect to miR-152 mimics transfection. The luciferase reporter analysis confirmed that miR-152 straightly targets the 3′-untranslated region (3’-UTR) of uncoupling protein 3 (*UCP3*) to cause its post-transcriptional inhibition in the protein level. The knockdown of *UCP3* by siRNA showed the similar effect of miR-152 on myofiber type transition. Furthermore, the rescue experiment in the porcine myotube transfected with miR-152 mimics or/and *UCP3* overexpression plasmid with or without the 3’UTR revealed that *UCP3* mediates the action of miR-152 in slow-twitch myofiber formation. Taken together, our findings proposed a novel molecular mechanism through which miR-152 epigenetically regulates meat quality via promoting slow-twitch myofiber formation and skeletal myogenesis.

## 1. Introduction

Meat quality characteristics of different pig breeds exhibit considerable differences because of distinct genetic selection processes [1,2,3]. As a result of this selection, the Rongchang (RC) pigs, a local fatty pig breed, have been developed for a poor growth rate and lower lean meat percentage, and superior meat quality [1,4]. In contrast, the large white (LW) pigs, a foreign lean pig breed, have a high growth rate and muscularity, but present inferior sensory quality of their meat [5]. Numerous studies have indicated that the differences in meat quality largely resulted from the differences in myofiber type compositions [6,7]. Therefore, the RC and LW pigs are ideal models to investigate the potential mechanisms for myofiber type differences in pork quality.

Skeletal muscle accounts for 40–50% of the total body weight and is composed of heterogeneous muscle fibers. According to the morphisms of the myosin heavy chain (MyHC), muscle fibers can be divided into four different types, including type I with *MyHC I*, type IIa with *MyHC IIa*, type IIx with *MyHC IIx*, and type IIb with *MyHC IIb* [8]. The type I and IIa fibers are slow and fast oxidative types, respectively. The type IIx and IIb fibers are intermediate and fast glycolytic fibers, respectively. In contrast with the type IIb fibers, the type I fiber has higher mitochondria and myoglobin content, lower myosin ATPase activity level and glycolytic capacity. Whereas the type IIb fiber, which has a larger fiber size, contains higher glycogen and shows more rapid contraction speed [3,9]. Numerous studies have demonstrated that muscle fiber characteristics are responsible for many aspects of meat quality, including meat color, tenderness, postmortem meat pH, water-holding capacity, intramuscular fat content, and flavor [10,11,12,13]. It was commonly believed that muscles containing a higher proportion of slow-twitch or oxidative fibers (*MyHC I* and *MyHC IIa*) led to the better meat quality than those with fast-twitch or glycolysis fibers [11,14,15].

The compositions of myofiber types can be influenced by a variety of endogenic and exogenous factors. Among them, microRNAs (miRNAs), endogenous non-coding RNAs, have been regarded as key modulators of myofiber specification [16,17]. Notably, the double knockout of miR-208b/miR-499, intronic miRNAs encoded by *Myh7* and *Myh7b*, showed an obvious slow-to-fast myofiber transition in mice [16]. Meanwhile, miR-133a, a muscle-specific miRNA, was reported to participate in skeletal myogenesis, mitochondrial function, and myofiber type conversion [18,19]. Most recently, Nielsen et al. reported that miR-152 is highly expressed in the skeletal muscle [20]. A previous study in the pig fetal LD muscle transcriptome discovered that miR-152 was more abundant in the LD muscle from Meishan pigs than that from the LW pigs [21]. Additionally, Li, et al. [22] revealed a key regulatory effect of miR-152 in the process of denervated fast muscle atrophy. Moreover, miR-152 was reported to regulate the proliferation and differentiation of *C2C12* myoblasts via targeting *E2F3* [23]. Interestingly, miR-152 has the same seed sequence as miR-148, which promotes myoblast differentiation through targeting *ROCK1* [24]. These results suggested that miR-152 has an underlying role in modulating porcine myofiber type compositions and myogenesis. However, it is still unclear whether miR-152 performs a direct role in porcine myofiber specification via targeting effector genes that mediate myofiber type transformation.

Uncoupling protein 3 (UCP3) is a transmembrane carrier protein located on the inner mitochondrial membrane and is expressed primarily and abundantly in the skeletal muscle [25]. It is believed as a key switch on energy expenditure through uncoupling oxidative phosphorylation independent of the ATP synthesis, leading to heat production [26,27]. Previous studies reported that UCP3 protein levels are lower in all fiber types of endurance-trained cyclists when contrasted with healthy controls, with a greater difference in the oxidative type I than in the glycolytic type II fibers [28]. Moreover, UCP3 protein is expressed the most in glycolytic type IIb fibers, more in oxidative-glycolytic type IIa fibers and the least in oxidative type I fibers [29]. In addition, physiological increases in UCP3 expression decreases the production of the reactive oxygen species (ROS) [30,31]. Many studies have shown that physiological concentrations of ROS are critical for maintaining normal cellular functions, such as cell growth, differentiation, proliferation, and apoptosis [32,33,34]. In the skeletal muscle, exercise can promote the formation of slow-twitch myofibers while causing the accumulation of intracellular ROS [35], which means a certain concentration of ROS may be beneficial to slow-fiber formation [36,37]. Therefore, these studies suggested that *UCP3* might perform a critical role in myofiber specification. However, no direct evidence was reported on a functional link between *UCP3* and myofiber type conversion.

The objective of our research is to clarify the potential influence of miR-152 on myofiber type transformation and skeletal myogenesis in pigs, and the underlying mechanism. Interestingly, we found that the miR-152 level is more abundant in the slow-twitch myofiber-enriched muscles, while the protein level of UCP3 was also reduced. Importantly, the gain- and loss-of-function experiments using synthetic miRNA mimics and inhibitor proved that miR-152 could promote slow-twitch myofiber formation and myogenesis in pigs. Mechanically, *UCP3* is validated as a direct target of miR-152 and can mediate its action in slow-twitch myofiber formation. Our findings provided a novel molecular target for ameliorating pork quality.

## 2. Materials and Methods

### 2.1. Animals and Sample Collection

The experimental protocol for the present research was reviewed and authorized by the Ethics Committee of the Southwest University of Science and Technology (DKX-1020150040, Mianyang, Sichuan, China) [38]. Five LW and five RC barrows of 160 days of age were selected and slaughtered in a humane manner. The following tissues, including heart, liver, spleen, lung, kidney, fat, soleus (SOL), gastrocnemius (GAS), psoas major muscle (PMM), longissimus dorsi muscle (LDM), and extensor digitorum longus (EDL), were collected and rapidly immersed in liquid nitrogen and preserved at −80 °C for further analysis.

### 2.2. Porcine Myoblasts Isolation and Culture

Primary porcine myoblasts were obtained from the LD muscle of two-day-old DLY (Duroc × Landrace × Yorkshire) piglets as described previously [39]. The harvested cells were identified by a flow cytometry with the Pax7 antibody (Invitrogen) (Supplementary Materials Figure S1). For the induction of myogenesis, the medium was switched to a differentiation medium containing DMEM/F12 (1:1) with 2% HS (Invitrogen) as porcine myoblasts grew to nearly 80% confluency.

### 2.3. Cell Transfection

Porcine myoblasts were induced to differentiate with a differentiation medium when cells grew to 80% confluency. Four days later, either the miR-152 mimics, mimics control, miR-152 inhibitor, inhibitor control, pcDNA3.1(+)-UCP3 (primer listed in Supplementary Materials Table S1), or siUCP3 (RiboBio) were transfected into porcine myotubes with a transfection reagent (L3000015, Invitrogen) following the manufacturer’s protocol. Then, at 24 h post transfection, the medium was shifted and porcine myotubes continued to culture for four days.

### 2.4. Real-Time PCR

The total RNA from tissue samples and porcine myotubes was isolated with a TRIzol reagent (9108Q, TaKaRa, Dalian, China) following the manufacturer’s suggestions. The yield and quality of RNA were evaluated with a spectrophotometer (Thermo, Waltham, MA, USA) and denatured gel electrophoresis. Reverse transcription was conducted to initiate the cDNA synthesis using a reverse-transcription kit (RR047A, TaKaRa, Dalian, China) with a specific stem-loop primer for miR-152 and oligo(dT) plus random primers for mRNAs. RT-PCR was conducted with the SYBR Green mix (RR820A, TaKaRa, Dalian, China) on a quantitative PCR machine (ABI Prism 7900HT, ABI, Carlsbad, CA, USA). The expression levels of miR-152 and myofiber development-related genes were analyzed utilizing the 2^−ΔΔCT^ method [40] with U6 and β-actin as references, respectively. The sequence of RT-qPCR primers and miRNA-specific stem-loop primers were listed in Table 1.

### 2.5. Western Blot Analysis

The total protein of muscle samples or adhered porcine myotubes were lysed in a round-bottom microcentrifuge tube using the RIPA lysis buffer (Beyotime, Jiangsu, China) at 4 °C for 10 min, and then homogenized with a homogenizer at 4 °C for 10 min [41]. After centrifugation, protein concentrations of lysates were detected by a BCA protein assay kit (Beyotime, Jiangsu, China). Before loading to the gel, protein lysates were denatured at 95–100 °C with the loading buffer for 10 min. The proteins were separated with SDS-PAGE and subsequently transferred to the PVDF membrane (Millipore, Eshborn, Germany). After immersing in a blocking solution (5% skim milk) for 1 h, the membranes were incubated with primary antibodies at 4 °C overnight and then with HRP-labeled secondary antibodies for 2 h at room temperature [42,43]. Primary antibodies specific for slow-MyHC (1:1000, Abcam, Shanghai, China), fast-MyHC (1:1000, Abcam), UCP3 (1:1000, Abcam), MyoG (1:200, Abcam), MyHC (1:1000, DSHB), α-Tubulin (1:3000, Beyotime, Beijing, China), and HRP-conjugated secondary antibodies (1:3000, Beyotime) were utilized to test protein expression. The ClarityTM Western ECL Substrate (Bio-Rad, Shanghai, China) was utilized to visualize protein blots. Blots were quantified by the Image Lab and normalized to α-Tubulin expression.

### 2.6. Luciferase Reporter Assay

To construct the UCP3 3’UTR luciferase reporter plasmid, the UCP3 3’UTR was amplified from porcine genomic DNA with the primer listed in Table S1. The purified PCR products were then inserted into the 3′-end of the Renilla luciferase reporter gene at the Xhol/NotI restriction sites of the psiCHECK-2 vector (Promega, Madison, WI, USA) and confirmed by sequencing (TsingKe Biotech, Chengdu, China). The site-directed mutagenesis was applied to introduce a six-base substitution into the miR-152-binding site of UCP3 3’UTR by mutagen primers listed in Table S1. For the luciferase reporter assay, HEK 293T cells were co-transfected with the psiCHECK-2 vector containing wt-UCP3 or mut-UCP3, as well as porcine miR-152 mimics or mimics control (Ribobio) using the Lipofectamine 3000 (Invitrogen). The medium was replaced 5 h after transfection. At 48 h post-transfection, the luciferase activity was measured with the Dual-Luciferase Reporter Assay System (Promega, Madison, WI, USA) following the manufacturer’s instructions. The relative luciferase activities were normalized to the Firefly luciferase activity.

### 2.7. Metachromatic ATPase Staining

ATPase staining was conducted as described previously [44]. In brief, LD muscle samples from LW and RC pigs were immersed in a cooled isopentane. Then, the transverse section was obtained, and subsequently incubated at pH 4.35 to identify the slow-twitch myofibers.

### 2.8. Metabolic-Related Enzymes Activities Analysis

Porcine myotubes were collected and homogenized in a cooled saline solution. Then, the activities of dehydrogenase (LDH), succinic dehydrogenase (SDH), and malate dehydrogenase (MDH) were detected with commercial kits (Nanjing Jiancheng Bioengineering Institute, Nanjing, China) [45].

### 2.9. Statistical Analysis

All data were analyzed using Microsoft Office Excel 365 and IBM SPSS statistics 20 version (SPSS Inc., Chicago, IL, USA). The results were presented as means ± S.E.M. Differences between groups were tested by Tukey’s test or one-way ANOVA. *p* < 0.05 were regarded as statistical significance.

## 3. Results and Discussion

### 3.1. MiR-152 is More Abundant in Slow-Twitch Myofiber-Enriched Muscles

**Figure 1:**
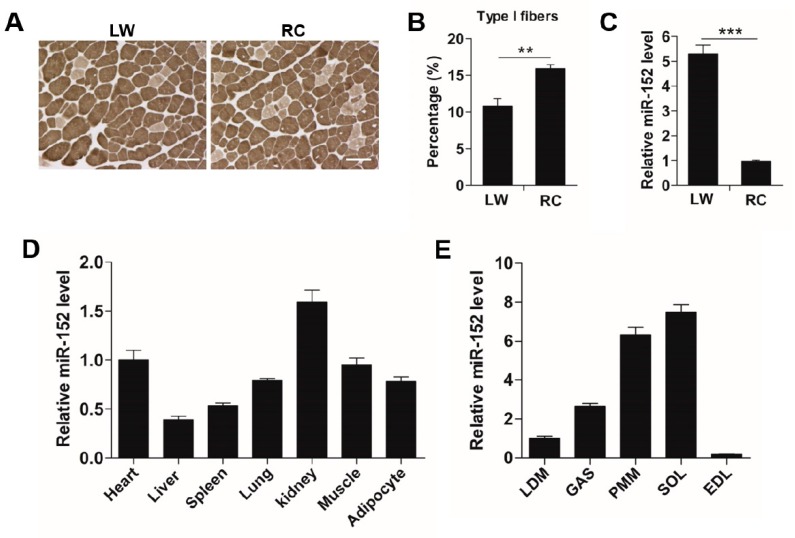
miR-152 is more abundant in the slow-twitch myofiber-enriched muscles. (**A**) Metachromatic adenosine triphosphatase (ATPase) staining of the longissimus dorsi (LD) muscles from large white (LW) and Rongchang (RC) pigs (*n* = 5, five pigs). Type I fibers stain light gray. Original magnification, ×100. Bars: 100 μm. (**B**) Proportion of the slow-twitch fiber was analyzed in the LD muscles from LW and RC pigs based on the ATPase staining. (**C**) miR-152 expression in the LD muscles from both LW and RC pigs was determined by RT-PCR (*n* = 5, five pigs). (**D**) Tissue distribution of miR-152 in adult LW pigs (*n* = 5, five pigs). (**E**) Expression of miR-152 in different types of skeletal muscle in adult LW pigs (*n* = 5, five pigs). Results are exhibited as mean ± S.E.M. ** *p* < 0.01 and *** *p* < 0.001 when contrasted with the control.

Different pig breeds exhibited considerable differences in pork quality characteristics [1,2,3], and these differences possibly resulted from the distinct myofiber types compositions in muscles [6,7]. Thus, the phenotype of myofiber in LD muscles from either LW or RC pigs were examined by metachromatic ATPase staining. As exhibited in Figure 1A,B, the proportion of slow-fiber in the LD muscle of the RC pigs was higher (more than 1.5-fold, *p* < 0.01) than that of the LW pigs, which was in line with the superior meat quality of RC pigs [1]. Intriguingly, previous research in the pig fetal LD muscle transcriptome discovered that miR-152 was more abundant in the LD muscle from Meishan pigs than that from the LW pigs [21], suggesting its potential role in myofiber development. Then, we compared the miR-152 level in LD muscles from either LW or RC pigs by RT-PCR. Consistent with previous reports, the miR-152 level was more abundant in the LD muscle of RC pigs (more than 5-fold, *p* < 0.01) when contrasted with that of the LW pigs (Figure 1C). In addition, the expression profile of miR-152 in various tissues showed that miR-152 was highly expressed in the heart, kidney, and skeletal muscle (Figure 1D), especially in slow-phenotype muscles, including PMM and SOL (Figure 1E). These consequences indicated that miR-152 is more abundant in the slow-twitch myofiber-enriched muscles.

### 3.2. MiR-152 Promotes Slow-Switch Myofiber Formation and Skeletal Myogenesis

**Figure 2:**
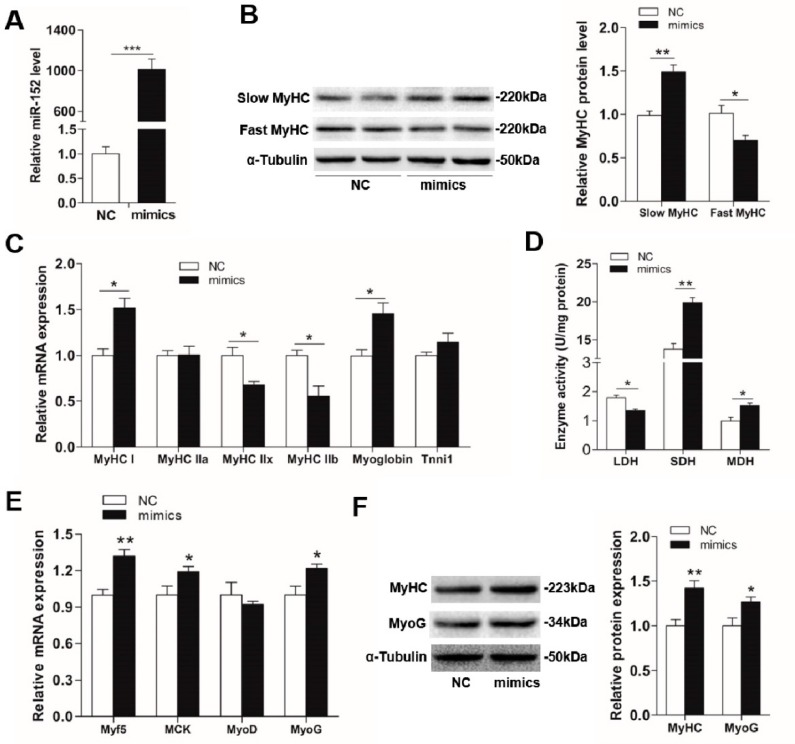
The influences of miR-152 mimics transfection on myofiber specification and skeletal myogenesis. (**A**) The miR-152 level was examined by RT-PCR at four days after transfection. (**B**) The protein expression of MyHC isoforms in porcine myotubes was detected at four days after transfection with miR-152 mimics. α-Tubulin served as an internal control. (**C**) *MyHC* isoforms and oxidative fiber markers were determined by RT-PCR. (**D**) The activities of metabolic-related enzymes in porcine myotubes transfected with miR-152 mimics. (**E**) Relative mRNA levels of *Myf5*, *MCK*, *MyoD*, and *MyoG* were detected by RT-qPCR at four days after miR-152 mimics transfection. (**F**) The protein expression of myogenic markers, MyHC, and MyoG, were detected by western blot at four days after miR-152 mimics transfection and analyzed using image J. Results are presented as mean ± S.E.M. (*n* = 4, four independent experiments). * *p* < 0.05, ** *p* < 0.01 and *** *p* < 0.001 when contrasted with the control.

**Figure 3:**
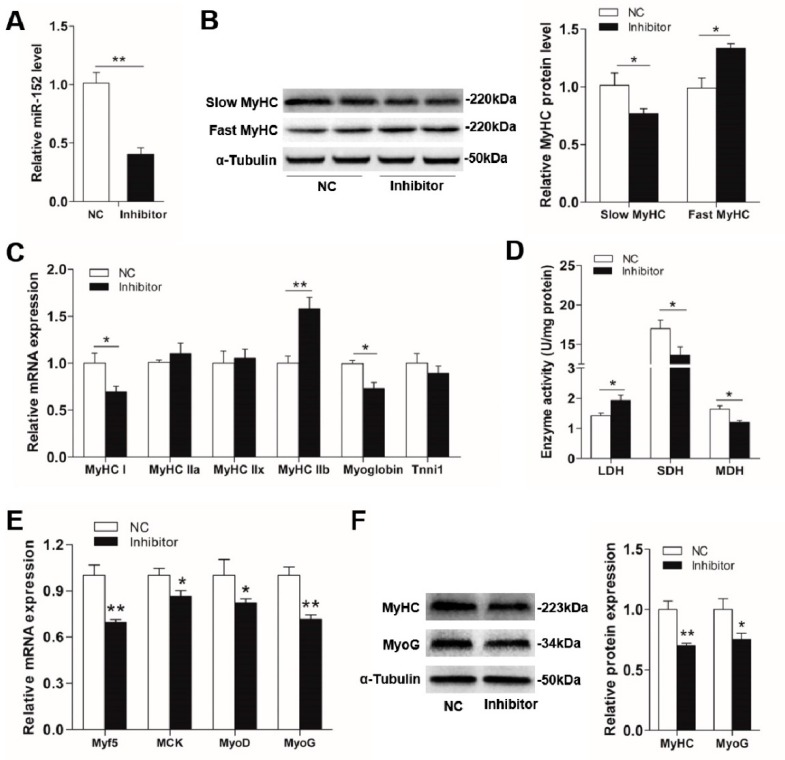
The effects of miR-152 inhibitor transfection on myofiber specification and skeletal myogenesis in porcine myotubes. (**A**) The miR-152 level was examined by RT-PCR at four days post transfection. (**B**) The protein expression of *MyHC* isoforms in porcine myotubes was detected at four days after transfection with the miR-152 inhibitor. α-Tubulin served as an internal control. (**C**) *MyHC* isoforms and oxidative fiber markers in porcine myotubes transfected with the miR-152 inhibitor were determined by RT-PCR. (**D**) Metabolic-related enzymes activities in porcine myotubes transfected with the miR-152 inhibitor. (**E**) Relative mRNA levels of *Myf5*, *MCK*, *MyoD*, and *MyoG* were detected by RT-qPCR at four days after miR-152 inhibitor transfection. (**F**) The protein expression of myogenic markers, MyHC and MyoG, were detected by western blot at four days after miR-152 inhibitor transfection and analyzed using image J. Results are exhibited as mean ± S.E.M. (*n* = 4, four independent experiments). * *p* < 0.05, ** *p* < 0.01 and *** *p* < 0.001 when contrasted with the control.

Previous studies reported that miR-152 was a key regulatory factor in the process of denervated fast muscle atrophy [22], and highly expressed in the skeletal muscle of Meishan pigs [21]. Meanwhile, miR-152 was reported to regulate the proliferation and differentiation of *C2C12* myoblasts through targeting *E2F3* [23]. However, whether miR-152 performs an important role in the porcine myofiber type transformation and myogenesis has not been explored. Given the differences of myofiber type compositions and miR-152 level between the LW and RC pigs, we hypothesized that miR-152 might be involved in myofiber specification and myogenesis. To investigate this hypothesis, miR-152 mimics, inhibitor, and respective controls were transfected into porcine myotubes. Four days after transfection, the miR-152 level in the mimics group was increased more than 1000-fold when contrasted with the group (Figure 2A, *p* < 0.001). Conversely, the miR-152 level in the inhibitor group was strikingly decreased (Figure 3A, *p* < 0.01). As exhibited in Figure 2B,F, the transfection of miR-152 mimics dramatically upregulated the protein expression of slow MyHC, MyHC, and MyoG, and downregulated the protein expression of fast MyHC, MyHC, and MyoG when contrasted with the control (*p* < 0.05). Moreover, overexpression of miR-152 increased the mRNA expressions of *MyHC I*, *myoglobin* (a marker of slow-twitch fibers), and myogenic transcriptional regulators (*Myf5*, *MCK*, and *MyoG*), as well as decreased the mRNA levels of *MyHC IIx*, and *MyHC IIb* (Figure 2C,E, *p* < 0.05). Nevertheless, the miR-152 inhibitor group exhibited the opposite effects to the miR-152 mimics group on myofiber specification and myogenesis (Figure 3B,C). These results directly showed that miR-152 could promote slow-twitch myofiber formation and skeletal myogenesis. However, there are some differences in the results of in vitro and in vivo. In the animal experiment, miR-152 expression was about 5-fold higher than that in LW and the percentage of the slow-twitch fiber was higher in RC by 40–50% compared to the LW muscles (Figure 1B,C). While, in porcine myoblasts, miR-152 expression was 1000-fold higher in the mimics transfected group, but the slow fiber was only elevated by 45–50% (Figure 2A,B). We speculated that the reason responsible for these differences may derive from differences in the internal and external environment, endocrine factor, and processing duration. However, it is worth noting that the trends of the myofiber type changes were consistent.

In addition, myofibers can be divided according to the energy metabolism features [46,47], and changes in the activities of metabolic-related enzymes can indirectly reflect alterations of myofiber types [48]. For instance, SDH and MDH, two oxidative-related enzymes, show higher activities in oxidized myofibers, while, LDH, a typical glycolytic-related enzyme, exhibits a higher activity in glycolytic myofibers [49]. As indicated in Figure 2D, overexpression of miR-152 promoted the activities of SDH and MDH and reduced the LDH activity when compared to the control, whereas opposite consequences were found in the miR-152 inhibitor group (Figure 3D), which indirectly supported the role of miR-152 in slow-twitch myofiber formation as described above.

### 3.3. UCP3 is a Direct Target of MiR-152

**Figure 4:**
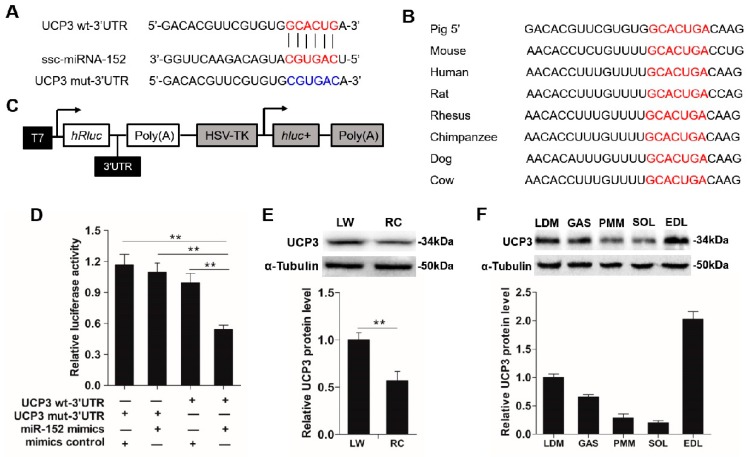
*UCP3* is a target of miR-152. (**A**) Schematic illustration of the predicted binding site for miR-152 in UCP3 3’UTR. The wild-type miRNA-binding sites in UCP3 3’UTR (red) were modified to complementary sequences (blue) to construct the mutated UCP3 3’UTR. (**B**) The predicted binding site of miR-152 in UCP3 3’UTR is highly conserved among mammals. (**C**) The sketch map of the dual-luciferase reporter vector psi-CHECK2. The putative miR-152 target site of UCP3 3’UTR and mutation target site were inserted into the psi-CHECK2 vector at the 3’ end of the *Renilla* luciferase gene (*hRluc*). The *Firefly* luciferase activity was treated as an internal normalization control. (**D**) The luciferase reporter containing the wild type and mutant UCP3 3’UTR was co-transfected into HEK293T cells with miR-152 mimics or mimics control. At 48 h post-transfection, the *Renillas* luciferase activity was measured and normalized to the *firefly* luciferase activity. (**E**) UCP3 protein expression in the LD muscles of LW and RC pigs (*n* = 5, five pigs), α-Tubulin served as the reference. (**F**) UCP3 protein expression in different types of skeletal muscle in adult LW pigs (n = 5, five pigs), α-Tubulin served as the reference. Results are presented as mean ± S.E.M. (*n* = 4, four independent experiments). * *p* < 0.05, ** *p* < 0.01 and *** *p* < 0.001 as contrasted with the control.

The above consequences showed that miR-152 can promote slow-twitch muscle fiber formation. Generally, miRNAs function in gene expression modification via combining with the 3’UTR of their target genes to induce messenger RNA degradation or translational repression [50]. To uncover the underlying mechanisms through which miR-152 modulates myofiber type transition, TargetScan and RNA22 were utilized to predict the feasible targets. Among which, the UCP3 3’UTR region had a conserved binding site with miR-152 (Figure 4A) across mammals from dogs to pigs (Figure 4B), which suggested that epigenetic modification may be a widespread pattern among mammals. Moreover, UCP3 is found to be related to the myofiber type transition and its protein expression differs among different fiber types [28,29,51]. Intriguingly, UCP3 protein level showed an inverse relation with miR-152 expression in the LD muscles from either LW or RC pigs (Figure 4E and Figure 1C). Meanwhile, we also observed that the expression level of miR-152 also exhibited an inverse correlates with UCP3 protein expression in different types of the skeletal muscle (Figure 1E and Figure 4F). These results indicated that UCP3 might be a potential target of miR-152 in regulating myofiber specification. To test whether *UCP3* was the target gene of miR-152, a fragment of pig UCP3 3’UTR with or without mutation at miR-152 binding regions was amplified and inserted into the 3’-end of *Renilla* gene in the psiCHECK-2 vector (Figure 4C). As expected, the luciferase activity was remarkably inhibited in HEK-293T cells when the psiCHECK-2 -UCP3-wt-3′UTR reporter was co-transfected with miR-152 mimics (Figure 4D, *p* < 0.01), whereas mutation of the miR-152 binding site in UCP3 3′UTR completely reversed this effect (Figure 4D, *p* < 0.01). These consequences indicated UCP3 is a target of miR-152.

**Figure 5:**
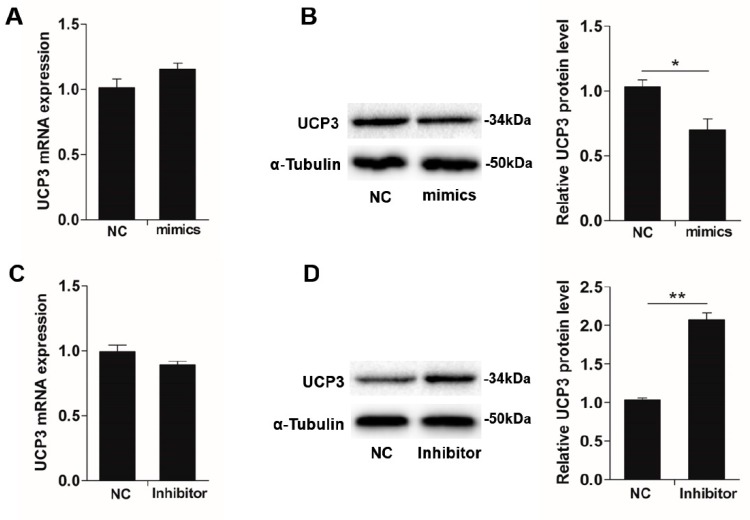
miR-152 downregulates *UCP3* expression in the protein level. *UCP3* expression in porcine myotubes transfected with miR-152 mimics were determined in mRNA (**A**) and protein (**B**) levels by qPCR and immunoblotting, respectively. UCP3 expression in porcine myotubes transfected with the miR-152 inhibitor were determined in mRNA (**C**) and protein (**D**) levels by RT-qPCR and immunoblotting, respectively. α-Tubulin served as the loading control. Results are exhibited as the mean ± S.E.M. (*n* = 4, four independent experiments). * *p* < 0.05 as contrasted with the control.

Since miR-152 could regulate myofiber type transformation and UCP3 was its target gene, we assumed the miR-152 level inversely correlates with UCP3 expression during slow-twitch myofiber formation. To confirm this hypothesis, UCP3 expression in the porcine myotube transfected with the miR-152 mimics and inhibitor were measured in mRNA and protein levels. Compliance with the hereinabove luciferase reporter analysis, miR-152 overexpression exhibited a noticeably inhibitory effect on the expression of endogenous UCP3 protein in the porcine myotube when contrasted to the mimic control (Figure 5B, *p* < 0.05), even if remarkable suppression was not checked at the mRNA level (Figure 5A, *p* > 0.05). By contrast, the miR-152 inhibitor transfection led to an increase in UCP3 protein nearly 2-fold (Figure 5D, *p* < 0.01). These data show that miR-152 promotes slow-twitch myofiber formation possibly via inhibiting UCP3 expression at the protein level.

### 3.4. UCP3 Mediates MiR-152 Action in Slow-Twitch Myofiber Formation

**Figure 6:**
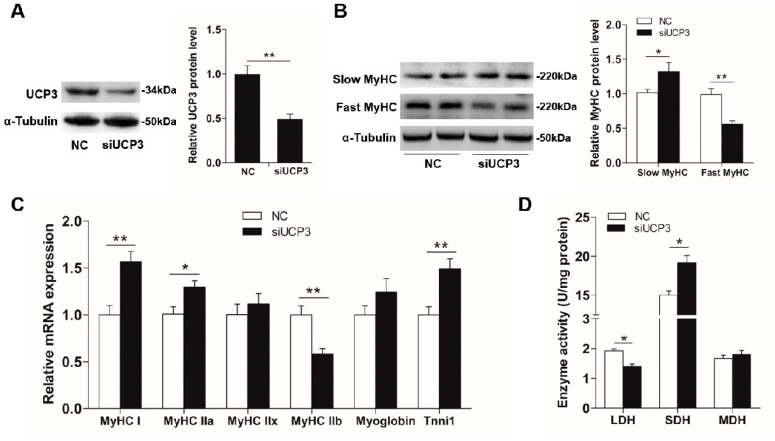
UCP3 promotes slow-to-fast myofiber type transformation. Transfection of siUCP3 into porcine myotubes knocked down *UCP3* expression in protein (**A**) levels, which led to slow-twitch myofiber formation (**B**) as determined by immunoblotting. (**C**) *MyHC* isoforms and oxidative fiber markers in porcine myotubes transfected with siUCP3 were determined by RT-PCR. (**D**) The activities of metabolic-related enzymes in porcine myotubes transfected with siUCP3. Results are exhibited as mean ± S.E.M. (*n* = 4, four independent experiments). * *p* < 0.05 and ** *p* < 0.01 when contrasted with the control.

It has been shown that UCP3 is related to muscle fiber specification and its protein expression differs among different myofiber types [28,29,51]. To investigate the role of UCP3 on myofiber type conversion, siUCP3 was transfected into porcine myotubes and its efficiency was verified using immunoblotting. As exhibited in Figure 6A, the protein level of UCP3 was remarkably reduced (2-fold, *p* < 0.01), when contrasted with the control, which proved that the silence of UCP3 in the porcine myotube was effective. In accordance with the role of miR-152 in myofiber type conversion, the silence of UCP3 dramatically promoted slow-twitch myofiber formation with an increase in slow MyHC protein expression (*p* < 0.05, Figure 6B) and the mRNA expression of *MyHC-I*, *MyHC-IIa*, and *Tnni1* (*p* < 0.01, Figure 6C), and a decrease in the protein expression of fast MyHC, as well as the mRNA expression of *MyHC-IIb* (*p* < 0.01, Figure 6B,C). The present data directly explained the previous observation that UCP3 expression was lower in oxidative fibers than that in glycolytic fibers [29,52,53]. Moreover, given the role of UCP3 in energy regulation, the activities of metabolic-related enzymes (LDH, MDH, and SDH) were also detected. As shown in Figure 6D, the knockdown of UCP3 significantly promoted the SDH activity and reduced the LDH activity (*p* < 0.05). Moreover, physiological increases in UCP3 expression decreases the production of reactive oxygen species (ROS) [30,31]. Although the ROS content was not detected in the present study, we speculated that the decrease of the ROS content might be the cause of myofiber type transformation. Studies have shown that physiological concentrations of ROS are critical for maintaining normal cellular functions, such as cell growth, differentiation, proliferation, and apoptosis [32,33,34]. In the skeletal muscle, exercise can promote the formation of slow-twitch muscle fibers while causing the accumulation of intracellular ROS [35]. It is therefore possible that a certain concentration of ROS may be beneficial to slow-fiber generation [36,37].

**Figure 7:**
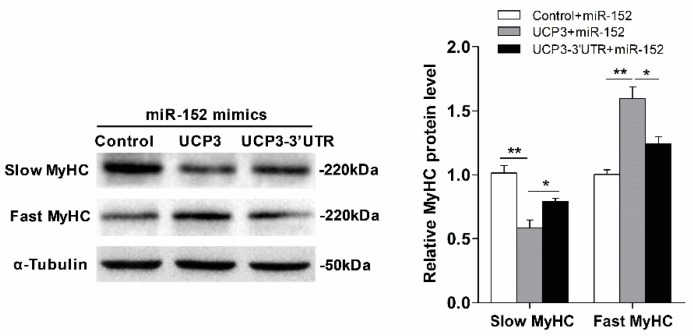
UCP3 mediates miR-152 action in slow-twitch myofiber formation. Protein expression of slow/fast MyHC in porcine myotubes co-transfected with miR-152 or/and *UCP3* overexpression plasmid with or without the 3′UTR. α-Tubulin served as an internal control. Results are exhibited as mean ± S.E.M. (*n* = 4, four independent experiments). * *p* < 0.05 and ** *p* < 0.01 when contrasted with the control.

To further investigate whether UCP3 mediates the miR-152 function in myofiber specification, a rescue trial was performed. As expected, overexpression of *UCP3* via the pcDNA3.1(+)-UCP3 vector without the 3’UTR remarkably reversed the action of miR-152 (Figure 7). Conversely, UCP3 with the 3’UTR relieved the above effect (Figure 7). Together, these data demonstrated that UCP3 conveys the action of miR-152 on slow-twitch myofiber formation.

## 4. Conclusions

In summary, our results revealed a critical role of miR-152 in myofiber specification and skeletal myogenesis. We found that miR-152 is more abundant in the slow-twitch myofiber-enriched muscles. Overexpression of miR-152 facilitated slow-twitch myofibers formation and myogenesis, whereas inhibition of miR-152 exhibited the opposite effect to the miR-152 mimics on myofiber type transformation and myogenesis. We further verified that miR-152 directly targets *UCP3* 3′UTR and remarkably decreased *UCP3* expression in the protein level. The knockdown of *UCP3* showed the same effect on myofiber specification as miR-152. Additionally, the rescue experiment in the porcine myotube transfected with the miR-152 or/and UCP3 overexpression vector with or without the 3′UTR uncovered that *UCP3* mediated the action of miR-152 in slow-twitch myofiber formation. Our findings implied that miR-152 and its target gene *UCP3* might be the underlying markers for interposition intended to promoting meat quality in domestic animals.

## Figures and Tables

**Table 1:** Primers for RT-qPCR.

Gene	Primer Sequence (5′ to 3′)	Accession No.
*MyHC I*	F: GTTTGCCAACTATGCTGGGG	NM_213855.1
	R: TGTGCAGAGCTGACACAGTC	
*MyHC IIa*	F: CTCTGAGTTCAGCAGCCATGA	NM214136.1
	R: GATGTCTTGGCATCAAAGGGC	
*MyHC IIx*	F: TTGACTGGGCTGCCATCAAT	NM_001104951.2
	R: GCCTCAATGCGCTCCTTTTC	
*MyHC IIb*	F: GAGGTACATCTAGTGCCCTGC	NM_001123141.1
	R: GCAGCCTCCCCAAAAATAGC	
*Myoglobin*	F: GGAAGGTGGAGGCTGATGTC	NM_214236.1
	R: CCGTGCTTCTTCAGGTCCTC	
*Tnni1*	F: AGGAACACGAGGAGCGAGAG	NM_213912.3
	R: CACCTTGGCGTGAAGTTCC	
*Myf5*	F: GAGTTCGGGGACGAGTTTGA	NM_001278775.1
	R: TTTCCTCTTGCACGCTTTGC	
*MCK*	F: CACCCCTTCATGTGGAACGA	NM_001129949.1
	R: TCGAACTTGGGATGCTTGCT	
*MyoD*	F: TGCCCAAGGTGGAAATCCTG	NM_001002824.1
	R: GCTGTAATAGGTGCCGTCGT	
*MyoG*	F: GAAAACTACCTGCCCGTCCA	NM_001012406.1
	R: CCACAGACACGGACTTCCTC	
*β-Acti* *n*	F: TGTACACACCTCATGCCAGC	EU655628.1
	R: GAGCCGCGTGTGTGTAACTA	
miR-152	RT: GTCGTATCCAGTGCAGGGTCCGAG GTATTCGCACTGGATACGACCCAAGT	
	F: CGCGACAGTGCATGACAGA	
	R: AGTGCAGGGTCCGAGGTATT	
*U6*	F: CTCGCTTCGGCAGCACA	
	R: AACGCTTCACGAATTTGCGT	

*MyHC*, myosin heavy chain; *Tnni1*, troponin 1; *Myf5*, myogenic factor 5; *MCK*, muscle creatine kinase; *MyoD*, myogenic differentiation factor; *MyoG*, myogenin.

## Supplementary Materials

The following are available online at https://www.mdpi.com/2076-2615/9/9/669/s1, Table S1: Primers used for plasmids construction. Figure S1: Identification of porcine primary myoblasts by flow cytometry with the Pax7 antibody.
