# Supplementary material for: MicroRNA-152 Promotes Slow-Twitch Myofiber Formation via Targeting Uncoupling Protein-3 Gene
Source: Animals (Basel). 2019 Sep 10;9(9):669. doi: 10.3390/ani9090669 (PMC6769457; doi:10.3390/ani9090669)
Supplement: Supplementary file 1 [file animals-09-00669-s001.zip › Supplementary materials/Table S1.docx]

**Table S1.** Primers used for plasmids construction

| **Items** | **Nucleotide Sequence of Primers (5'–3')** |
| --- | --- |
| UCP3 wt-3′UTR | F: CCGCTCGAGGTAAGACAAAGCCACCTGGC |
|  | R: ATTTGCGGCCGCAAATGTTAGGCATCCATCCTAGTG |
| UCP3 mut-3′UTR | F: CGTGACcaagatgctcaataaattatattaatttgtgaaa |
|  | R: cacacgaacgtgtcctgaaggtgaagcaccggagacca |
| pcDNA3.1(+)-UCP3 | F: CCCAAGCTTATGGTGGGCCTGAAGCCT |
|  | R: CCGCTCGAGTCAAAACGGAGATTCCCGCA |
| pcDNA3.1(+)-UCP3-3′UTR | CCCAAGCTTAGGGCACTCGGGTCCCTG |
|  | CCGCTCGAGAAATGTTAGGCATCCATCCTAGTG |

“______” are restriction enzyme cutting sites; “ ” are mutated sequences.
