# Supplementary figures and images for: MicroRNA-152 Promotes Slow-Twitch Myofiber Formation via Targeting Uncoupling Protein-3 Gene
Source: Animals (Basel). 2019 Sep 10;9(9):669. doi: 10.3390/ani9090669 (PMC6769457; doi:10.3390/ani9090669)

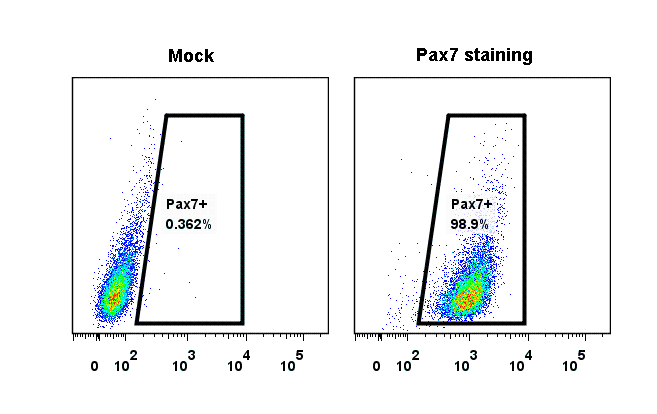
**Fig 1.** Identification of porcine myoblast by flow cytometry with Pax7 antibody

Supplement: Supplementary file 1 [file animals-09-00669-s001.zip › Supplementary materials/Figure S1.docx]
